# Entrepreneurship education on entrepreneurial intention: The moderating role of the personality and family economic status

**DOI:** 10.3389/fpsyg.2022.978480

**Published:** 2022-09-23

**Authors:** Yiran Liu, Min Li, Xin Li, Jingyi Zeng

**Affiliations:** School of Education, Tianjin University, Tianjin, China

**Keywords:** entrepreneurship education, entrepreneurial intention, proactive personality, narcissistic personality, family economic status

## Abstract

This study investigates the impact of entrepreneurship education on college students’ entrepreneurial intentions, as well as the moderating effects of personality and family economic status on the relationship between entrepreneurship education and entrepreneurial intention, respectively. We tested our hypotheses using a sample of college students in Tianjin, China, and analyzed the data of 326 questionnaires containing validated measures. The results show that entrepreneurship education has a positive impact on college students’ entrepreneurial intentions; proactive personality negatively moderates this relationship; and family economic status positively moderates it. However, the moderating effect of narcissistic personality has not been verified. This study is unique and innovative as it brings new insights to this stream of literature by introducing the roles of the personality and family economic status in the relationship between entrepreneurship education and entrepreneurial intention. Our analysis provides important empirical evidence about the negative moderating effect of proactive personality and the positive moderating effect of family economic status on the relationship between entrepreneurship education and entrepreneurial intention, introducing insights into the heterogeneity of the effect of entrepreneurship education.

## Introduction

Given that China’s economy has transitioned from a capital- and factor-driven economy to an innovation-driven one, innovative and entrepreneurial talents are in high demand (Jian et al., 2021). In this context, encouraging college students to start their own businesses is a critical approach for enhancing China’s innovation development and economic transformation (Lv et al., 2021). Although entrepreneurship is highly uncertain, and entrepreneurs’ characteristics differ greatly, it is not impossible to teach entrepreneurship. College students are more likely to become entrepreneurs in the future if they acquire entrepreneurial knowledge and skills through entrepreneurship education (Hahn et al., 2019; Lv et al., 2021). Thus, entrepreneurship education has received much attention from the government and universities, and how to evaluate and develop entrepreneurship education has become an important issue in the field of education (Daneshjoovash and Hosseini, 2019).

Entrepreneurial intention is regarded as an important antecedent in driving entrepreneurial behavior (Fayolle and Liñán, 2014). There will be no entrepreneurial activities if there are no entrepreneurial intention (Bird, 1988). After years of research, entrepreneurship education has been proven to facilitate students’ entrepreneurial intentions (Zhao et al., 2005; Souitaris et al., 2007). Entrepreneurial intention is also regarded as a critical indicator of the effect of entrepreneurship education (Daneshjoovash and Hosseini, 2019). Existing research indicates that entrepreneurship education encourages college students to acquire entrepreneurial knowledge and skills and alters their ways of thinking (Souitaris et al., 2007). Entrepreneurship education can also motivate students to gain a more comprehensive understanding of entrepreneurship, which improves their entrepreneurial self-efficacy and opportunity recognition ability (Zhao et al., 2005; Karlsson and Moberg, 2013; Nowiński et al., 2019), influencing entrepreneurial intention indirectly (Hoang et al., 2020). However, it remains unknown why some students even after receiving entrepreneurship education, continue to have low entrepreneurial intentions. It is necessary to investigate the factors that influence the heterogeneity of the effects of entrepreneurship education.

The effects of education are determined by the characteristics of the individuals who are educated (Ganzach and Gotlibovski, 2014), such as their personalities (Williamson, 2021). Many studies emphasize the significance of personality in entrepreneurship (Ciavarella et al., 2004; Hisrich et al., 2007; Fairlie and Holleran, 2012; Basuki et al., 2021). Among these, proactive and narcissistic personalities have attracted increasing attention in academia (Bateman and Crant, 1993; Liu et al., 2019; Neneh, 2019). Existing literature indicates that individuals with high level of proactive personality are persistent and take more initiative (Hu et al., 2018). They are better at identifying and capitalizing on opportunities and, as a result, are more likely to start a new business (Neneh, 2019). Narcissistic individuals have higher level of self-esteem and exaggerated self-worth (Judge et al., 2006). They crave affirmation and appreciation from others in order to strengthen their self-image (Wallace and Baumeister, 2002). Entrepreneurship provides narcissists with a context in which they can reinforce their elevated ego (Navis and Ozbek, 2017). As a result, narcissism increases entrepreneurial intention (Hmieleski and Lerner, 2016; Gao and Huang, 2021) and promotes entrepreneurial entry (Wales et al., 2013). However, it remains unclear how this relationship interacts with external factors such as education to influence entrepreneurial intention. Although these two personalities have been shown to be very important to entrepreneurship, we hypothesize a negative moderating effect, since individuals with high level of proactive personality are willing to achieve the goals and acquire useful information initiatively before being educated. Thus, they are hard to be influenced by entrepreneurship education. And for highly narcissists, they pay more attention to self-views and inner world so that they tend to ignore the guidance and suggestions of others, even of entrepreneurship education.

Family economic status also plays a key role in improving entrepreneurial intention by offering financial security (Rusu et al., 2022). Most students lack financial capital and resources, which creates obstacles to entrepreneurial entry (Wright et al., 2006). Seeking assistance from families has become an important approach to solving financial problems for entrepreneurs, especially youth (Sieger and Minola, 2017; Rusu et al., 2022). However, there has been little quantitative analysis that how family economic status influences the effect of entrepreneurship education. Not all families can provide sufficient entrepreneurial funds, whose economic status matters. Through getting entrepreneurial knowledge, students become aware of the economic risks and financial pressures inherent in entrepreneurship. Those from poor families may be concerned that starting businesses will become a burden on their families, thus decreasing entrepreneurial intention. Therefore, we propose that family economic status positively moderates the relationship between entrepreneurship education and college students’ entrepreneurial intentions.

Starting with the baseline hypothesis that entrepreneurship education increases entrepreneurial intention, this study explores the factors that impact the effects of entrepreneurship education on entrepreneurial intention. We test our hypothesis with a sample of 326 college students in Tianjin, who have accepted entrepreneurship education. Our study empirically shows that proactive personality negatively regulates the effect of entrepreneurship education on entrepreneurial intention while family economic status positively regulates this relationship.

This study has several contributions. First, this study adds to research on the mechanism of the influence of entrepreneurship education on entrepreneurial intention. By investigating the moderating role of proactive and narcissistic personalities and family economic status, we shed light on the types of students who can be easily educated and how to make entrepreneurship education more effective Second, previous studies have shown that individuals with high level of proactive personality are more likely to establish a new firm because they are characterized as innovation, exploration and initiative (Crant, 1996; Becherer and Maurer, 1999; Neneh, 2019). However, we find out its dark side that people with high level of proactive personality are difficult to absorb external opinions and guidance. They are hard to be taught and educated. Third, we highlight the significance of family economic foundation in entrepreneurship. Most college students acquire start-up funding from home because they lack sufficient income to support their businesses (Elston et al., 2016). This study argues that poor families may induce timidity, even for students with increased entrepreneurial knowledge and improved entrepreneurial ability. Economic risks to families make them afraid to start a business.

This study addresses a gap in the literature by examining the relationship between entrepreneurship education, entrepreneurial personalities, family economic status, and entrepreneurial intention. It also responds to calls to assess the impact of entrepreneurship education on students’ personal factors (Sun et al., 2020).

## Literature review and hypothesis development

### Entrepreneurial intention

Entrepreneurial behaviors are motivated by entrepreneurial intention (Bird, 1988). Without entrepreneurial intention, there would be no subsequent entrepreneurial action (Zhang and Huang, 2021). Therefore, high level of entrepreneurial intention effectively predict entrepreneurial entry (Fayolle and Liñán, 2014). Bird (1988) was the first to propose this concept and defined entrepreneurial intention as a mental state that makes a person invests much attention, energy, and time to achieving a specific goal. Starting with the definition, Krueger stated that entrepreneurial intention is the commitment of potential people to implement entrepreneurial activities in the future (Krueger, 2007). Individuals with greater entrepreneurial intention are more likely to establish their own firms after graduation (Krueger, 2007). Some scholars even believe that entrepreneurial intention is the belief that a person intends to start a new venture (Thompson, 2009). In this study, we define entrepreneurial intention as a person’s subjective tendency and psychological preparation for establishing a new venture (Krueger, 2007; Bae et al., 2014; Esfandiar et al., 2019).

### Entrepreneurship education

The concept of entrepreneurship education was first proposed by the United Nations Educational, Scientific and Cultural Organization at the “International Symposium on Education for the 21st Century,” which was held in Beijing in 1989. Colin Bohr, an OECD expert, stated that entrepreneurship education develops and improves students’ basic entrepreneurial qualities and entrepreneurial abilities, ensuring that they have the necessary knowledge, abilities, and psychological qualities to engage in entrepreneurial activities. He considered entrepreneurship education to be “the third educational passport.” Entrepreneurship education is a kind of practical education that cultivates many innovative talents with basic literacy in entrepreneurship and continuously injects new power into the innovation and entrepreneurship of the country (Hahn et al., 2019; Lv et al., 2021).

Existing research indicates that entrepreneurship education can positively impact entrepreneurial intentions. According to an American study of MBA students, the number of students who have taken entrepreneurial management courses is positively related to entrepreneurial intentions (Sagie and Elizur, 1999). A study on British and French college students majoring in science and engineering also showed that attending entrepreneurial classes and training has a positive impact on students’ entrepreneurial intentions (Souitaris et al., 2007). Entrepreneurship education inspires students to their entrepreneurial ideas into specific, concrete actions (Souitaris et al., 2007). Cultivation of entrepreneurial ability through entrepreneurship education has a significant effect on one’s decision to start a business (Burke et al., 2002). Moreover, entrepreneurship education also plays an important role in shaping the inner power of the “entrepreneurship spirit.” In summary, receiving entrepreneurship education is conducive to stimulating college students’ entrepreneurial inspiration (Nabi et al., 2018), accumulating entrepreneurial knowledge, and cultivating entrepreneurial abilities and skills (Solomon et al., 2019; Muñoz et al., 2020), making it easier for them to enhance their entrepreneurial intentions and participate in entrepreneurship. Therefore, this study proposes the following hypothesis:

*H*1: Entrepreneurship education is positively correlated with college students’ entrepreneurial intentions.

### Proactive personality

Personality characteristics affect an individual’s ability and creativity at work (Morgeson et al., 2005). People tend to choose occupations that match their personality characteristics (Miller and Miller, 2005). The concept of proactive personality was first proposed by Bateman and Crant (1993), who believed that it is a behavioral tendency that is not restricted by environmental resistance and takes the initiative to change the environment. Individuals with high level of proactive personality are good at identifying and exploring opportunities, as well as show characteristics of being active and persistent until meaningful changes occur (Hu et al., 2018). They usually act ahead of time and keep going until their expectations are met. In contrast, people with low level of proactive personality have less initiative to seek new information and inactively control their environment (Bateman and Crant, 1993). Entrepreneurial spirits, including innovativeness, proactiveness and high risks (Covin and Slevin, 1991), are highly consistent with proactive personality. Studies have shown that a person with a high level of proactive personality is more compatible with entrepreneurial behavior and activities (Crant, 1996; Becherer and Maurer, 1999; Neneh, 2019).

Although entrepreneurship education increases college students’ entrepreneurial intentions, it may be different for students with higher level of proactive personality. They are usually not satisfied with the knowledge of their major and actively dabble in knowledge in various fields (Major et al., 2006). In the context of “mass entrepreneurship and innovation in China, there are numerous ways to gain entrepreneurship knowledge. Students with high level of proactive personality may be more likely to acquire knowledge in the field of entrepreneurship on their own rather than through entrepreneurship education. Hence, for students with high level of proactive personality, the impact of entrepreneurship education on knowledge accumulation and ability improvement is significantly reduced. Furthermore, individuals with high level of proactive personality exhibit more assertiveness, which means they tend to stick to their own opinions (Hu et al., 2018), making them less likely to be educated. In contrast, students with low level of proactive personality rarely learn about new things by themselves. Entrepreneurship education can be viewed as a means of introducing new entrepreneurial knowledge to them and broadening their horizons, thereby increasing their entrepreneurial intentions. Therefore, this study proposes the following hypothesis:

*H*2: Proactive personality negatively regulates the relationship between entrepreneurship education and college students’ entrepreneurial intentions. The higher the level of proactive personality, the weaker the relationship between entrepreneurship education and entrepreneurial intention.

### Narcissistic personality

Nowadays, narcissism is no longer considered a personality disorder but a kind of normal personality in common people (Raskin and Terry, 1988), which is primarily characterized by self-focus, self-centeredness, or selfishness (Lasch, 1980; Harms et al., 2020). People with high level of narcissistic personality exaggerate their self-worth and think that they are more talented than others (Judge et al., 2006). Meanwhile, they are eager to succeed and keen to accept admiration from others in order to strengthen their self-image (Wallace and Baumeister, 2002). Entrepreneurship is a good way to achieve self-superiority. Many studies have shown that narcissism is significantly positively correlated with entrepreneurial intentions and entry (Mathieu and St-Jean, 2013; Hmieleski and Lerner, 2016; Gao and Huang, 2021).

Individuals with high level of narcissistic personality are self-centered, arrogant, and unwilling to accept the opinions and suggestions given by others (Emmons, 1987; Wink and Donahue, 1997). The self-centered nature of narcissists makes entrepreneurship education less effective. On the contrary, people with low level of narcissistic personality will participate in entrepreneurship education with a more open attitude, thus forming a deeper understanding of entrepreneurship and generating stronger entrepreneurial intentions. Therefore, this study proposes the following hypothesis:

*H*3: Narcissistic personality negatively moderates the relationship between entrepreneurship education and college students’ entrepreneurial intentions. The higher the level of narcissism, the weaker the relationship between entrepreneurship education and entrepreneurial intention.

### Family economic status

High uncertainty is one of the characteristics of entrepreneurship (McMullen and Shepherd, 2006), and it also necessitates the acquisition of initial funds by entrepreneurs (Calic and Mosakowski, 2016). Research has shown that the family is an important source of start-up capital (Elston et al., 2016), especially for young college students (Rusu et al., 2022). The implementation of entrepreneurship education will assist students in acquiring corresponding entrepreneurial knowledge, allowing them to gain a preliminary understanding of the capital needs and risks involved in the entrepreneurship process. After learning about the high capital investment and the high failure rate of entrepreneurship, students with low family economic status may hesitate or give up the idea of starting a business because they do not have sufficient funding. On the one hand, it’s difficult for poor families to put a large amount of money into entrepreneurship. On the other hand, if the business fails, their families may be in debt, which is a huge burden for the family undoubtedly. However, students with a high family economic status will not be restricted by funds for entrepreneurship. Financial support from the family encourages them to start their own businesses without fear, even though they are aware of the risks. Therefore, this study proposes the following hypothesis:

*H*4: Family economic status positively moderates the relationship between entrepreneurship education and college students’ entrepreneurial intentions. The higher the family’s economic status, the stronger the relationship between entrepreneurship education and entrepreneurial intention.

**Figure 1:**
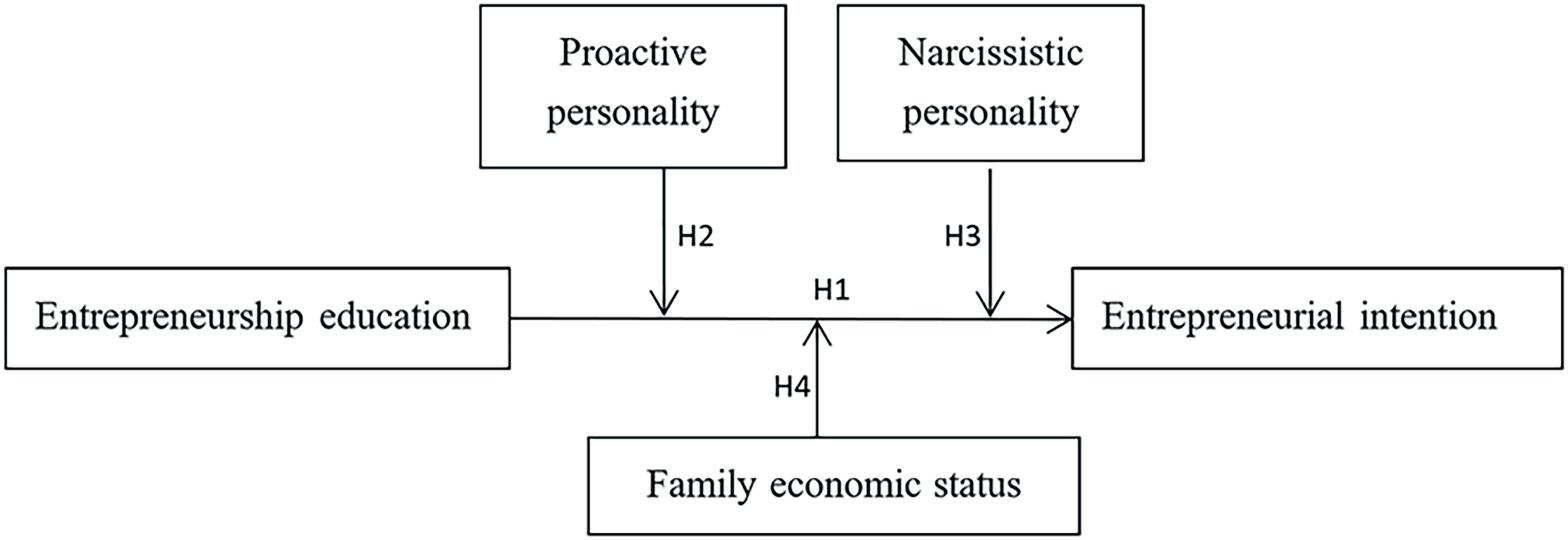
The conceptual research model.

The study’s research model is comprised of the four hypotheses listed above (see Figure 1).

## Methodology

### Sample and data collection

We conducted the survey in Tianjin, China. As the calling of “mass entrepreneurship and innovation,” colleges and universities in China are paying increasing attention to cultivating students’ entrepreneurial spirits by developing entrepreneurship education. As an important economic center in North China, Tianjin is abundant in higher education resources, with numbers of various levels and types of colleges and universities. Tianjin has also created a favorable environment for college students to start businesses by implementing entrepreneurship education in colleges and universities, thereby injecting innovative and entrepreneurial forces into local and national development.

In this study, we surveyed college students in Tianjin, China. Questionnaires were distributed randomly among the students from different levels and types of colleges and universities, trying our best to follow the principle of stratified sampling through an online platform. The survey lasted for 20 days, from December 2019, and 332 questionnaires were returned. After excluding six questionnaires because of missing values on variables or completely identical values in one item, 326 valid questionnaires remained, yielding an effective rate of 98.19%.

The demographics of the samples were analyzed using SPSS 22.0, and the results are shown in Table 1. Among the respondents, males accounted for 52.8%; among the academic year, sophomores accounted for 41.7%; science and engineering majors accounted for 65.3%; families with 50–100 thousand RMB a year accounted for 37.7%.

**Table 1:** Sample demographics.

Variables	Category	Frequency	Percentage (%)
Gender	Male	172	52.8
	Female	154	47.2
Academic year	Freshman	33	10.1
	Sophomore	103	41.7
	Junior	70	21.5
	Senior	101	31.0
	the Fifth-year	1	0.3
	Other Year	18	5.5
Major	Science and Engineering Major	213	65.3
	Economic and Management Major	20	6.1
	Agronomy and Medicine Major	9	2.8
	Humanities and Social Sciences Major	72	22.1
	Other Major	12	3.7
Annual Household Income(RMB)	<50 thousand	82	25.2
	50–100 thousand	123	37.7
	100–200 thousand	63	19.3
	200–300 thousand	34	10.4
	300 thousand – 1million	15	4.6
	>1 million	9	2.8
Parental Entrepreneurial Experience	Both	25	7.7
	Either	38	11.7
	Neither	263	80.7

*N = 326*.

### Measurement of variables

#### Entrepreneurial intention

We used the Entrepreneurial Intention Scale compiled by Chen et al. (1998), which includes five items, such as “I am very interested in starting a business” and “I have made sufficient preparations for starting a business.” The 7-point Likert scale was used to measure entrepreneurial intentions, with 1 indicating completely not matched and 7 indicating completely matched. A higher score indicates a higher level of entrepreneurial intention. The Cronbach’s α for this scale was 0.926.

#### Entrepreneurship education

In this article, entrepreneurship education primarily refers to the course training, lectures, and practice related to entrepreneurship that students receive at universities. We designed three items to assess entrepreneurship education: “How many semesters of entrepreneurship-related courses have you taken?,” “How many lectures on entrepreneurship have you attended?” and “How many times have you attended entrepreneurship practice training?” (Kong and Zhao, 2017). The mean of the three responses is used to explain the level of entrepreneurship education. The higher the mean, the higher the level of education. The Cronbach’s α for this scale was 0.669.

#### Proactive personality

We used the Proactive Personality Scale developed by Seibert et al. (1999), which contained 10 items, such as “I have been constantly looking for new ways to improve my life throughout my life,” “No matter where I am, I am the main character for constructive change.” The 7 – point Likert scale was used to measure the level, with 1 indicating completely not matched and 7 indicating completely matched. The higher the score, the higher the level of proactive personality. Cronbach’s α for this scale was 0.890.

#### Narcissistic personality

We used the Narcissistic Personality Inventory-16 items (NPI-16) compiled by Ames et al. (2006), such as “(a) I know that I am excellent because people keep telling me that, (b) I sometimes feel cramped when others praise me;” “(a) I like to be the center of attention, (b) I prefer to follow the crowd.” Each question required respondents to choose one item from (a) and (b), with a = 1 and b = 0. The higher the score, the higher the level of narcissistic personality. Cronbach’s α for this scale was 0.828.

#### Family economic status

Economic status is a concept that cannot be directly measured (Bollen et al., 2007), but can be measured by proxy variables, such as income and expenditure (Cuc and Griffin, 2007). In this study, we used annual household income to measure family economic status. Based on the 2015 Chinese annual household income classification standard, the annual household income level was divided into six degrees: 1 = less than 50 thousand RMB (about 75 hundred US dollars), 2 = 50–100 thousand RMB, 3 = 100–200 thousand RMB, 4 = 200–300 thousand RMB, 5 = 300 thousand–1 million RMB, and 6 = more than 1 million RMB. The higher the income level, the higher the family’s economic status.

#### Control variables

Other factors included basic personal and family information. Previous studies have demonstrated that the entrepreneurial intentions of males are higher than those of females (Nowiński et al., 2019), and that the entrepreneurial intentions of students majoring in engineering are higher than those of business majors (Kolvereid and Moen, 1997; Gilmartin et al., 2019). Family members’ entrepreneurial experience influences entrepreneurial intentions (Aldrich and Cliff, 2003; Zhang and Huang, 2021). Entrepreneurial intentions are also influenced by the academic year (Yan and Ye, 2009). The control variables in this study were gender, academic year, major, and parental entrepreneurial experience.

### Concerns for common method variance

As the data came from a single survey, common method variance may exist in the measurement due to the proximity of the timing, medium, or location in which respondents participate, as well as similarities, sensitivity, or ambiguity in the wording of items (Podsakoff et al., 2003; Edwards, 2008). Therefore, we used the unmeasured latent factor technique to address this concern. First, we constructed a confirmatory factor analysis model, and the results of the main indicators were RMSEA = 0.058, SRMR = 0.0696, CFI = 0.861, and TLI = 0.850. The second model was constructed by adding an unmeasured latent factor based on the original. By comparing the results of the two models (△RMSEA = 0.008 < 0.05; △SRMR = 0.0156 < 0.05; △CFI = 0.044 < 0.05; △TLI = 0.041 < 0.05), it can be concluded that common method variance did not affect the study’s result.

### Confirmatory factor analysis

To ensure the discriminant validity of the study’s five key variables, we used the structural equation modeling software AMOS 24.0 to test the fit indices. The results showed *χ*2/df = 1.461 < 3, SRMR = 0.067 < 0.80, CFI = 0.939 > 0.90, TLI = 0.931 > 0.90, and RMSEA = 0.038 < 0.08, indicating that the indicators could meet the ideal standard (Hu and Bentler, 1999). The results are presented in Table 2.

**Table 2:** Results of confirmatory factor analysis.

Model fit	*χ*^2^/df	RMSEA	CFI	IFI	TLI	PNFI
Standard	<3	<0.08	>0.9	>0.9	>0.9	>0.5
Results	1.461	0.038	0.939	0.940	0.931	0.740

## Results

### Correlation analysis

We used SPSS 22.0 to analyze the mean value, standard deviation, and correlation coefficients among the variables in this study, and the summary statistics and correlations are displayed in Table 3. The correlations between entrepreneurship education and entrepreneurial intention was positive and significant (*r* = 0.126, *p* < 0.05). The correlation between proactive personality and entrepreneurial intention was positive and significant (*r* = 0.187, *p* < 0.01). The correlation between narcissistic personality and entrepreneurial intention was positive and significant (*r* = 0.247, *p* < 0.01). The correlation between family economic status and entrepreneurial intention was positive (*r* = 0.079) and marginally significant.

**Table 3:** Correlation coefficient matrix among variables.

	1	2	3	4	5	6	7	8	9
Entrepreneurial Intention	1								
Gender	0.096	1							
Academic year^a^	−0.048	0.121^*^	1						
Parental entrepreneurial experience^b^	0.138^*^	0.027	0.065	1					
Major^c^	0.030	−0.065	0.059	0.004	1				
Entrepreneurship education	0.126^*^	0.139^*^	0.221^**^	−0.034	0.115^*^	1			
Narcissistic personality	0.247^**^	0.105	−0.038	0.102	−0.122^*^	−0.032	1		
Proactive personality	0.187^**^	−0.004	−0.011	−0.034	−0.064	0.102	−0.213^**^	1	
Annual household income	0.079	0.160^**^	−0.022	0.223^**^	0.051	−0.003	0.193^**^	0.083	1
Mean	3.180	0.52	2.96	0.193	0.061	1.026	0.396	3.466	2.40
Standard deviation	1.509	0.500	1.242	0.395	0.240	1.055	0.254	0.679	1.250

*N* = 326.

* *p* < 0.05;

** *p* < 0.01.

a Academic year coded as Freshman = 1, Sophomore = 2,Junior = 3,Senior = 4, the Fifth-year = 5, Other Year = 6.

b Parental entrepreneurial experience coded as Both = 1, Either = 2, Neither = 3.

c Major coded as Science and Engineering Major = 1,Economic and Management Major = 2,Agronomy and Medicine Major = 3,Humanities and Social Science Major = 4,Other Majors = 5.

### Hypothesis testing

In this study, we test the hypotheses with a hierarchical regression analysis. Results are shown in Table 4. Model 1–5 are separate models, and Model 6 is full model. Model 1 includes control variables and entrepreneurial intention. Model 2 adds entrepreneurship education. Model 3–5 include each regulating variable, and interaction term of entrepreneurial intention with them, respectively. Model 6 is the full model which includes all the variables.

**Table 4:** Regression results of entrepreneurial intention on each variable.

	Model 1	Model 2	Model 3	Model 4	Model 5	Model 6
Gender	−0.082	−0.064	−0.070	−0.046	−0.051	−0.046
Academic year	−0.070	−0.109^+^	−0.103^+^	−0.096^+^	−0.112^*^	−0.098^+^
Parental entrepreneurial experience	−0.129^*^	−0.146^**^	−0.147^**^	−0.117^*^	−0.155^**^	−0.143^**^
Major	−0.053	−0.054	−0.053	−0.034	−0.068	−0.048
Entrepreneurship education		0.152^**^	0.137^*^	0.147^**^	0.151^**^	0.137^*^
Proactive personality			0.241^***^			0.207^**^
Entre edu * PP			−0.116^+^			−0.127^+^
Narcissistic personality				0.228^***^		0.205^**^
Entre edu * NP				−0.020		−0.012
Annual household income					−0.064	−0.117
Entre edu * AHI					0.128^+^	0.143^+^
*R* ^2^	0.031	0.052	0.088	0.096	0.060	0.131
Adjusted *R*^2^	0.019	0.037	0.068	0.076	0.040	0.101
△*R*^2^	0.031	0.021	0.008	0.000	0.008	0.020
*F*	2.550	3.481	4.369	4.827	2.925	4.310
*N*, df	326.4	326.5	326.7	326.7	326.7	326.11

+ *p* < 0.1;

* *p* < 0.05;

** *p* < 0.01;

*** *p* < 0.005.

*N* = 326. Entre edu represents Entrepreneurship education; PP represents Proactive personality; NP represents Narcissistic personality; AHI represents Annual household income.

Hypothesis 1 proposes that entrepreneurship education has a positive effect on entrepreneurial intention. In Model 2, the coefficient estimate of entrepreneurship education is positive and statistically significant (*β* = 0.152, p < 0.01), and Hypothesis 1 is supported.

**Figure 2:**
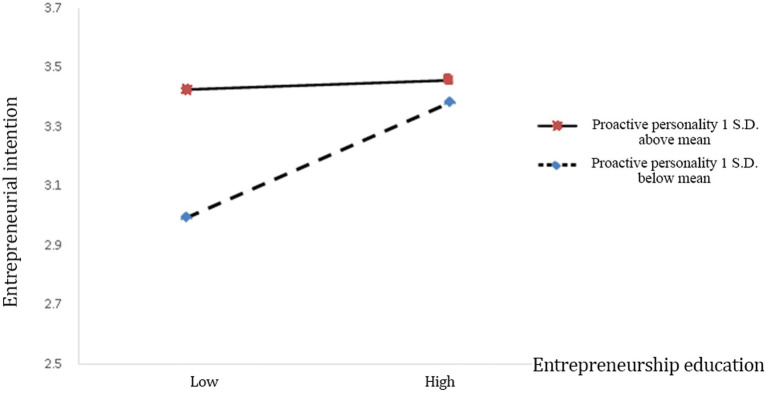
The negative moderating role of proactive personality on the relationship between entrepreneurship and entrepreneurial intention.

Hypothesis 2 proposes that proactive personality negatively moderates the relationship between entrepreneurship education and entrepreneurial intention. In Model 3, the interaction term of entrepreneurial intention and narcissistic personality is is negative and statistically significant (*β* = −0.116, *p* < 0.1). Therefore, Hypothesis 2 is supported, and Figure 2 depicts this relationship.

**Figure 3:**
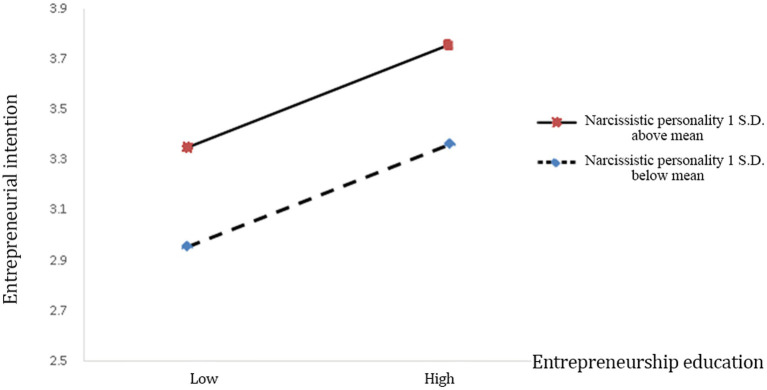
The effect of narcissistic personality on the relationship between entrepreneurship education and entrepreneurial intention.

Hypothesis 3 proposes that narcissistic personality negatively moderates the relationship between entrepreneurship education and entrepreneurial intention. In Model 4, the interaction term of entrepreneurial intention and narcissistic personality is negative but insignificant (*β* = −0.020, *p* > 0.1). Therefore, Hypothesis 3 is not supported and Figure 3 depicts this result.

**Figure 4:**
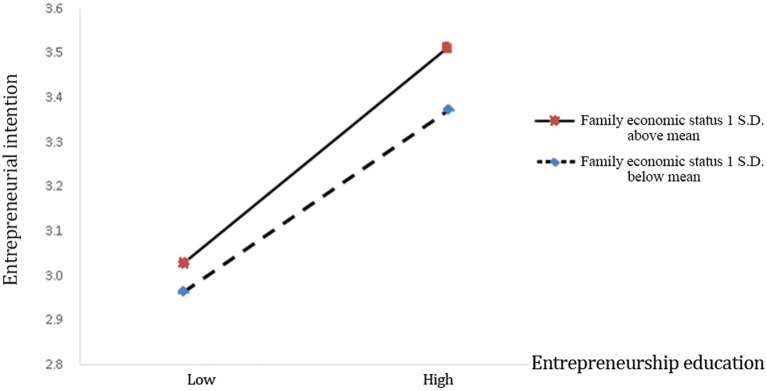
The positive moderating effect of family economic status on the relationship between entrepreneurship education and entrepreneurial intention.

Hypothesis 4 proposes that family economic status positively moderates the relationship between entrepreneurship education and entrepreneurial intention. In Model 5, the interaction term of entrepreneurship education and annual household income is positive and statistically significant (*β* = 0.128, *p* < 0.1). Accordingly, Hypothesis 4 is supported, and Figure 4 illustrates this relationship.

According to Model 6, the results of the full model are consistent with separate models that Hypothesis 1,2 and 4 are supported while Hypothesis 3 is not supported.

## Conclusion and discussion

### Conclusion

This study empirically explores the boundary conditions of the relationship between entrepreneurship education and entrepreneurial intention by investigating the moderating effects of the personality and family economic status. By collecting 326 valid questionnaires from college students in Tianjin, this study empirically analyzed the data and drew conclusions. First, entrepreneurship education has a positive and significant effect on entrepreneurial intention, which is consistent with previous research (Bae et al., 2014; Nowiński et al., 2019; Hoang et al., 2020; Lv et al., 2021). This finding highlights the importance of entrepreneurship education.

Second, proactive personality negatively moderates the relationship between entrepreneurship education and entrepreneurial intention. However, the negative moderating effect of narcissistic personality on entrepreneurship education and intention has not been verified. Previous studies have shown that proactive personality is one of the most critical characteristics of entrepreneurs (Becherer and Maurer, 1999; Neneh, 2019). But in this study, we reveal its negative side that people with high level of proactive personality hard to be influenced by entrepreneurship education to start new businesses. They show a tendency that they are more likely to start a business but difficult to absorb others’ advice and guidance directly.

Third, this study demonstrates that family economic status plays a positive moderating role in the relationship between entrepreneurship education and entrepreneurial intention. Previous studies have found that entrepreneurial family background plays an important role in moderating the effect of entrepreneurship education, emphasizing the role model created by entrepreneurial family members in this process (Lee et al., 2021). Further, our analysis emphasizes the importance of the economic part of the family background.

### Discussion

#### Theoretical implications

First, while previous studies have shown that entrepreneurship education promotes entrepreneurial intention (Hahn et al., 2019; Lv et al., 2021), this study empirically explored the moderating mechanism of this relationship by revealing the negative role of proactive personality and the positive role of family economic status. Both entrepreneurship education (Souitaris et al., 2007; Solomon et al., 2019; Muñoz et al., 2020) and the important entrepreneurial characteristics (Becherer and Maurer, 1999; Fairlie and Holleran, 2012; Neneh, 2019; Basuki et al., 2021) are the driving forces of entrepreneurial intention. This study suggests that these two driving forces interact in a negative way. In doing so, we also shed light on the heterogeneity in the effect of entrepreneurship education.

Second, our study illuminates how education affects students with proactive personalities. Previous studies have found that proactive personality is positively related to learning performance (Major et al., 2006) and academic engagement (Chen et al., 2021). Students with high level of proactive personalities show stronger learning motivation (Major et al., 2006) and achieve better school performance (McNall and Michel, 2011; Tymon and Batistic, 2016). However, our study provides evidence that students with high level of proactive personality are less likely to be educated despite their high level of entrepreneurial intention (Neneh, 2019). This suggests that proactive students are better able to learn on their own. Thus, this study contributes to our understanding of why students with high level of proactive personality perform better than others.

Third, this study points out that family economic status affects entrepreneurship education, which contributes to the literature on family background and entrepreneurship. While previous studies have primarily focused on the positive side of family background, such as role modeling by entrepreneurial family members (Lee et al., 2021; Zhang and Huang, 2021), this study focuses on the negative aspects. Entrepreneurship education will improve students’ understanding of entrepreneurship, but it also means that students will realize the high risks and investment of entrepreneurship, which requires a good family economic foundation (Rusu et al., 2022). This study argues that poor families may induce timidity, even for students with increased entrepreneurial knowledge and improved entrepreneurial ability.

#### Practical implications

An increasing number of colleges and universities are aware of the importance of entrepreneurship education and are successfully improving students’ entrepreneurial intentions. However, some students are still unwilling to start a business after receiving entrepreneurship education. Thus, how to further improve the impact of entrepreneurship education and what aspects can help solve this problem have become important practical topics (Yang et al., 2021).

Considering the various personalities of students, colleges and universities must provide more targeted and flexible entrepreneurship education. Specifically, schools could assess students’ personalities and recommend that they participate in the education that works for them. Students with high level of proactive personality are inclined to get new information and take actions forward so that schools can encourage them to attend entrepreneurial projects and activities rather than traditional class teaching. Give more freedom for them to achieve goals through their own efforts, rather than teach them how do to strictly. Furthermore, students with high level of narcissism are eager to start new businesses to enhance their self-satisfaction and gain attention from others. Entrepreneurial competition could be set up by giving high praise and sufficient rewards to participants in order to stimulate their entrepreneurial enthusiasm and passion.

While colleges and universities have taken the lead in developing students’ entrepreneurial abilities and skills, the government is responsible for providing economic support. Innovative and entrepreneurial talent are important forces for regional economic development and innovation-driven growth in China. Through entrepreneurial knowledge, college students become aware of the importance of financial capital, particularly those from low-income families. They desperately need financial assistance from the government because their families are unable to afford entrepreneurial funds. Therefore, we provide the following suggestions: (a) increasing the amount of an entrepreneurial loan; (b) appropriately lowering the tax for start-ups; and (c) reducing the repayment interest in a period over time in order to break down entrepreneurial barriers caused by students’ family circumstances.

## Limitation and future research direction

Entrepreneurship education encompasses both entrepreneurial theory and practice, which were not separately assessed in this study but have the potential to inspire future research. For instance, students with high level of proactive personality may perform better in practical situations than in classes focused on entrepreneurial theory. Therefore, while paying more attention to the role of students’ personalities, future research could explore the effects of entrepreneurship education using different educational methods.

## Data availability statement

The raw data supporting the conclusions of this article will be made available by the authors, without undue reservation.

## Ethics statement

The authors abide by academic ethics and morality. All respondents stated that the data in the questionnaires were available for research.

## Author contributions

YL, JZ, and ML proposed the research topic and designed the research framework. JZ and XL collected and analyzed the data and drew tables and figures. YL and ML drafted the manuscript and made several important revisions. As the corresponding author, ML ensured that all the authors approved the submission of the final version.

## Funding

Data collection was supported by a grant of the National Natural Science Foundation of China (71902136) and a grant by China’s Ministry of Education (18YJC630107).

## Conflict of interest

The authors declare that the study was conducted without any direct or latent commercial relationships that could be interpreted as potential conflicts of interest. All authors contributed to the article and approved the submitted version.

## Publisher’s note

All claims expressed in this article are solely those of the authors and do not necessarily represent those of their affiliated organizations, or those of the publisher, the editors and the reviewers. Any product that may be evaluated in this article, or claim that may be made by its manufacturer, is not guaranteed or endorsed by the publisher.
